# More Liver Metastases Detected Intraoperatively Indicates Worse Prognosis for Colorectal Liver Metastases Patients after Resection Combined with Microwave Ablation

**DOI:** 10.1155/2022/3819564

**Published:** 2022-04-21

**Authors:** Ling-Min Jiang, Yuan-Ping Zhang, Chen-Wei Wang, Wei-Dong Zhang, Wei He, Ji-Liang Qiu, Yi-Chuan Yuan, Bin-Kui Li, Yun-Fei Yuan, Ren-Chun Lai, Dan-Dan Hu, Yun Zheng

**Affiliations:** ^1^Department of Liver Surgery, Sun Yat-Sen University Cancer Center, Guangzhou, China; ^2^State Key Laboratory of Oncology in South China and Collaborative Innovation Center for Cancer Medicine, Sun Yat-Sen University Cancer Center, Guangzhou, China; ^3^Department of Radiology, Sun Yat-Sen University Cancer Center, Guangzhou, China; ^4^Department of Anesthesiology, Sun Yat-Sen University Cancer Center, Guangzhou, China

## Abstract

**Background:**

Whether more tumor numbers detected in surgery compared to preoperative image affecting survival of colorectal liver metastases (CRLM) patients after hepatectomy combined with microwave ablation (MWA) remains unclear.

**Methods:**

From 2013 to 2018, 85 CRLM patients who underwent hepatectomy combined with MWA were retrospectively assessed. Compared to the tumor numbers in preoperative image, patients with equal intraoperative tumor numbers were defined as the equal number group (*n* = 45); patients detected more tumor numbers in surgery were defined as the more number group (*n* = 40). Clinicopathological factors and prognosis were compared between two groups.

**Results:**

Compared to the equal number group, the more number group was characterized by more lymphatic metastasis, synchronous metastasis of liver lesion, and tumor numbers over 5 (all *P* < 0.05). Median survival time was 46.7 months and 26.8 months in the equal and more number group. Significantly worse overall survival (OS) was found in more number group to the equal number group (*P* = 0.027). In Cox analysis, more tumor number than image and high level of carbohydrate antigen 19-9 (CA19-9) were poor prognostic factors for OS.

**Conclusion:**

In patients receiving hepatectomy combined with MWA, detecting more liver metastases in surgery than preoperative image indicates poor long-term survival. These patients were characterized by more lymphatic metastasis, synchronous metastasis of liver lesion, and tumor numbers over 5. Intensive follow-up to detect early recurrence and potent postoperative therapy to improve survival may be justified in patients detected more tumor numbers in surgery with a high CA19-9 level.

## 1. Introduction

Colorectal cancer (CRC) is one of the most commonly diagnosed cancer worldwide with a rising incidence for now [1, 2]. The liver is the most common metastatic site for CRC, which present in approximately 20% of patients at the time of diagnosis, and nearly 60% of the rest patients during the course of the disease [3]. Hepatic resection offers the best chance for long-term survival in colorectal liver metastases (CRLM) patients, but only about 25% CRLM present with resectable disease [4–6].

Microwave ablation (MWA), one of the curative-intent treatment modalities of thermal ablation techniques, provides acceptable local tumor control rate with minimal morbidity and represents a treatment alternative for CRLM [7, 8]. Moreover, the combination of MWA and hepatectomy enlarges the candidate for surgery [9, 10]. With the guidance of intraoperative ultrasonography (US), MWA is broadly used in patients lack of reserved liver parenchyma or with liver lesions located near the vital structures [11].

In daily clinical practice, more hepatic lesions can be detected intraoperatively than preoperative image in some patients. Whether patients with more tumor numbers detected intraoperatively than preoperative image have different clinicopathological characteristics and prognosis compared to those with equal liver lesions remained unclear. In this study, we aimed to investigate the clinical value of detected more tumor number in surgery in CRLM and provide our evidence for better treatment options regarding these patients.

## 2. Materials and Methods

### 2.1. Patients

From November 2013 to October 2018, CRLM patients who underwent hepatectomy combined with intraoperative MWA at our center were reviewed from database. A total of 85 pathologically confirmed CRLM patients were retrospectively included. All patients received primary tumor resection prior to or combined with hepatectomy. The inclusion criteria were as follows: Eastern Cooperative Oncology Group-performance status (ECOG − PS) ≤ 2; Child-Pugh score of A or B, and no history of other malignancies. Exclusion criteria consisted of the following: double primary malignancies; extrahepatic metastasis; and incomplete clinical data or lost in follow-up. Compared to the tumor numbers in preoperative image, patients with equal intraoperative tumor numbers were defined as the equal number group (*n* = 45); patients detected more tumor numbers in surgery were defined as the more number group (*n* = 40). Differences in clinicopathological characteristics and prognosis between two groups (more number group vs. equal number group) were compared. This study was conducted in accordance with the Helsinki Declaration, and the Sun Yat-sen University Cancer Center ethics committee approved this study. Written informed consent was obtained before treatment.

### 2.2. Preoperative Management

Preoperative image used in the assessment of liver metastases by magnetic resonance imaging (MRI) or computed tomography (CT) was carried out within 2 weeks before resection of liver lesions. Before resection, all patients were evaluated with thoracoabdominal and pelvic imaging (US, CT, and/or MRI) to stage the disease. Positron emission tomography (PET)/CT was used to exclude extrahepatic disease for those with a suspect. Preoperative blood tests which included tumor markers were carried out within 2 weeks before resection of liver lesions. Intraoperative US with a linear probe with 3.5-4 MHz transmit and receive center frequency was performed in all patients.

### 2.3. Treatment of Liver Lesions

Hepatic lesions were resected when they were diagnosed as liver metastasis based on any of the preoperative or intraoperative image evaluation and palpation in surgery. If the resection of any lesion was unfeasible for safety reasons, MWA was conducted under IOUS guidance. According to the manufacturer specifications, all procedures were performed by inserting the probe within the lesion and maintaining a power of 40-80 W for an ablation time of 0.5-20 min. All ablated lesions are less than 3 cm. Complete ablation zone was considered optimal when a safety margin of at least 5 mm was obtained [12]. Additional ablation is performed if the complete coverage of the lesion zone was not reached. Track ablation was performed by slowly drawing back the MWA probe during the last 10 s of ablation to avoid needle tract implantation metastases.

### 2.4. Follow-Up

All patients were followed up monthly in the first 3 months, every 3 months in the first two years, and every 3 to 6 months thereafter. Physical examination, blood tests, and abdominal and pelvic US or CT/MRI were used for the surveillance of recurrence as appropriate. Definition of R0 resection is resection of liver lesions with clear histological margins, and non R0 (R1/R2) resection is resection with histological positive margins or residual lesions in intra or extrahepatic. Liver metastases diagnosed before, during or within 3 months after colorectal resection, are defined as synchronous metastases; while others are defined as metachronous metastasis. The clinical risk score (CRS) used in the study was an established risk score, which is consisting of five clinical factors, including primary lymph node metastasis, synchronous metastases, multiple liver tumors, tumor size over 5 cm, and carcinoembryonic antigen (CEA) over 200 ng/ml [13]. Each of these clinical factors is assigned 1 point. Patients with a CRS of 0–2 were regarded as the low-risk subgroup, while patients with a CRS of 3–5 were regarded as the high-risk subgroup.

### 2.5. Statistical Analysis

The primary endpoint of this study was overall survival (OS). The OS was defined as the time interval from liver resection to death from any cause or the last follow-up date. The recurrence-free survival (RFS) was defined as the time interval from liver resection to disease recurrence, death from disease, or the last follow-up date. Independent-sample *T*-test, Chi-square test, or Fisher's exact test were used for analyzing the differences in clinicopathological characteristics between two groups as appropriate. The OS and RFS curves were constructed by the Kaplan–Meier method and compared with the log-rank test. The Cox proportional hazard regression model was performed to identify the hazard ratio (HR) of prognostic factors. A *P* value <0.05 was considered statistically significant. All *P* values of statistical tests in the present study were two-sided. All statistical calculations were performed with the IBM SPSS Statistics 25.0 software package (SPSS Inc., Chicago, IL).

## 3. Results

### 3.1. Clinicopathological Characteristics

The baseline clinicopathological and laboratory parameters of the patients are listed in Table 1. According to the tumor numbers detected in preoperative image and intraoperative US, 45 (52.9%) patients were classified in the equal number group; 40 (47.1%) patients were classified in the more number group. Compared to the equal number group, the more number group presented with more lymphatic metastasis (81.6% vs. 50.0%, *P* = 0.005) and synchronous metastases of CRLM (90.0% vs. 68.9%, *P* = 0.017). No significant difference was shown in tumor numbers detected in preoperative image between two groups (median, 5 vs. 5, *P* = 0.700). There were 29 (72.5%) and 17 (37.8%) patients with over 5 hepatic tumor numbers detected intraoperatively in the more number and equal number group, respectively (*P* = 0.001). Twenty-five (62.5%) patients in the more number group and 15 (33.3%) patients in the equal number group had a CRS over 2 (*P* = 0.007). Other baseline parameters such as CEA over 200 ng/ml (2.6% vs. 6.7%, *P* = 0.392) and carbohydrate antigen 19-9 (CA19-9) over 35 kU/L (25.6% vs. 28.9%, *P* = 0.739) were comparable between two groups.

### 3.2. Survival Analysis

**Figure 1:**
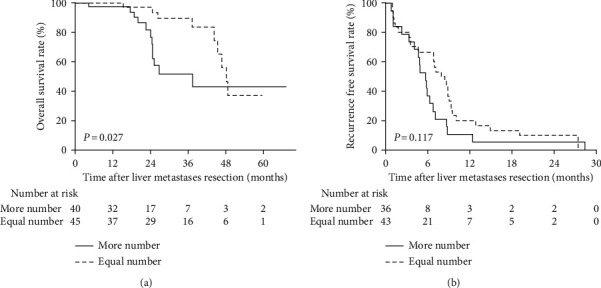
Overall survival (a) and recurrence-free survival (b) in the more number group and the equal number group.

The median OS for the more number group and the equal number group was 26.8 months and 46.7 months, respectively. The 1-, 3-, and 5-year OS rates after CRLM resection were 97.4%, 51.8%, and 43.1% in the more number group and 97.0%, 89.6%, and 37.2% in the equal number group, respectively. The more number group had a significantly worse OS than the equal number group (*P* = 0.027; Figure 1(a)). The 1- and 2-year RFS rates after R0 resection combined with complete ablation of liver metastases in the more number group were 5.3% and 0% and 16.7% and 10% in the equal number group. The RFS was comparable between two groups (*P* = 0.117; Figure 1(b)).

### 3.3. Prognostic Factors for Patients after Hepatectomy Combined with MWA

Next, we performed univariate and multivariate analysis to identify prognostic factors in our patients. Factors including high CA19-9 level (HR 4.29, 95% confidence interval [CI] 1.77-10.37, *P* = 0.001) and more number detected intraoperatively than preoperative image (HR 3.19, 95% CI 1.28-7.97, *P* = 0.013) were found to be independently associated with shorter OS (Table 2). Factors including tumor number detected in surgery over 5 (HR 1.90, 95% CI 0.78-4.63, *P* = 0.158) and tumor size over 5 cm (HR 4.36, 95% CI 0.96-19.7, *P* = 0.056) were not affecting long-term outcomes. For RFS, significant factor in multivariate analysis was tumor number over 5 detected in surgery (HR 1.95, 95% CI 1.07-3.56, *P* = 0.030).

**Figure 2:**
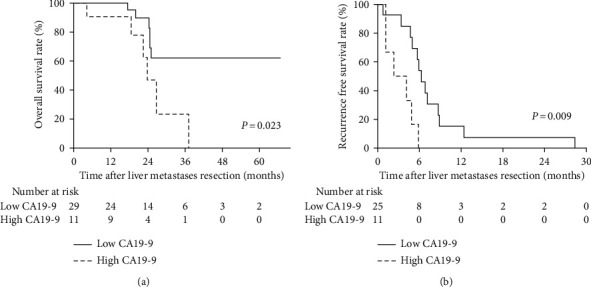
Overall survival (a) and recurrence-free survival (b) in the more number group patients with low CA19-9 level and high CA19-9 level.

A further multivariate Cox proportional hazard model was used to determine prognostic factors in the more number group patients. As shown in Table 2, high CA19-9 level was the only significant predictive factor for both OS and RFS in the more number patients. To evaluate the prognostic value of preoperative CA19-9 levels in these patients, survival curves were constructed by the Kaplan–Meier method and compared by the log-rank test. In the more number group, patients with high CA19-9 levels had significantly poor OS (*P* = 0.023) and RFS (*P* = 0.009) than those with low CA19-9 levels (Figure 2).

## 4. Discussion

With the better understanding of the CRLM, implication of new treatment modality, such as hepatic arterial infusion (HAI) and hepatectomy combined with ablation, and more potent chemotherapy regimens have improved the survival of patients [14–16]. The identification of prognostic factors will help to establish and optimize therapeutic strategies for CRLM. Many clinicopathological factors and molecular features are reported to affect survival of CRLM patients [17–19]. Among them, multiple tumor numbers in the liver have been associated with poor survival and more recurrence after resection [20, 21]. With the guidance of intraoperative US, more hepatic lesions can be detected in surgery than preoperative image in some patients. Compared to tumor numbers, the clinical value of detecting more tumor numbers intraoperatively than preoperative image remains unclear. The present retrospective study analyzed the data of 85 CRLM patients who underwent hepatic resection with MWA. Our results demonstrate that more tumor numbers detected in surgery are independently indicating worse long-term survival in CRLM patients receiving hepatectomy combined with MWA. We noted that the 5-year OS rate was higher in the more number group compared to the equal number group. Two patients in the more number group survived over five years and small simple size in our study resulted in the high 5-year survival rate in the more number group. However, considering the effect in all patients for survival by means of statistics, detected more tumor numbers in surgery is significantly correlated with shorter OS in CRLM patients.

A number of factors including sensitivity of imaging techniques and the biology of individual tumor may jointly contribute to the tumor number difference between intraoperative findings and preoperative evaluation. Regarding imaging studies, intraoperative US may better detect intrahepatic lesions compared to preoperative CT or MRI. Meanwhile, small metastases adjacent to the liver capsule may be discovered by inspection or palpation. Early study reported that large tumors can be well-illustrated by preoperative image, while small foci may be overlooked [22]. Moreover, tumor biological behavior may contribute to more liver metastases in surgery than preoperative image. Though the complex interplay between cancer cells and their microenvironment constituents remains to be elusive, it is acknowledged that gut microbiota promotes tumor metastasis in the colorectal cancer [23–25]. Disease progression during the waiting period indicates nonresponse to preoperative chemotherapy and more aggressive malignant feature. No response to preoperative chemotherapy is also negatively affecting survival in CRLM patients [26, 27].

Besides investigating the clinical value of detecting more tumor numbers in surgery, we also analyzed the clinical features of these patients. Patients in the more tumor number group tend to have more lymphatic metastasis, synchronous metastasis of liver lesion, and tumor numbers over 5. High CA19-9 level in these patients is associated with poor survival than patients with low CA19-9 level. Besides in patients detected more tumor numbers intraoperatively, high level of CA19-9 was also an independent predictor of poor OS in patients with colorectal liver oligometastases [28]. Therefore, in some subgroup of CRLM patients, level of CA19-9 may be a crucial marker for evaluation of prognosis. These novel findings together suggest that patients detected more tumor numbers in surgery with high CA19-9 level may need to receive intensive preoperative chemotherapy to eliminate micrometastatic disease and, more importantly, to further identify aggressive disease and select appropriate candidates for surgery. Besides, some elements could inhibit colon cancer cells' proliferation and enhance the valid outcomes of chemotherapy [29, 30]. Additionally, the benefit of surgery and high risk of recurrence should be carefully taken into consideration. Since subsequent treatments are crucial for prolonging survival of patients after recurrence, more frequent follow-up after surgery to detect early recurrence may help improve the prognosis of these patients.

Early study reported that total number of liver lesions is an important prognostic factor for survival [31, 32]. But tumor number was not an independent prognostic factor for OS in this study. In addition, there were also studies indicated that tumor number was not affecting survival in their cohort [33–35]. We speculate that the predictive value of a factor can vary among different populations. Thus, in CRLM patients after treatment of resection combined with MWA, more number detected in surgery may have more profound value than tumor number itself.

Our study has several limitations. First, it is a retrospective study with limited number of patients in a single institution. Validation in prospective, multicenter, and large scale of patients is necessary. Second, we only included patients who underwent hepatectomy with MWA. More specific studies which include patients receiving resection combined with radiofrequency ablation are needed to confirm our conclusion. Third, the reason why CA19-9 is a negative factor in the more number group was unclear. As a gastrointestinal cancer, it is closely related to dietary factors. The intake of foods, like Allium and its constituents, is significantly related to modify the malignancy of colon cancer [36]. Whether Allium extracts could potentially influence the cause of colorectal liver metastases still needs to be further investigated. Future molecular biology experiments are essential to explain the underlying mechanism behind this indicator.

## 5. Conclusions

In patients receiving hepatectomy combined with MWA, detected more liver metastases in surgery than preoperative image indicates worse long-term survival. These patients were characterized by more lymphatic metastasis, synchronous metastasis of liver lesion, and tumor numbers over 5. Intensive follow-up to detect early recurrence and potent postoperative therapy to improve survival may be justified in patients detected more tumor numbers in surgery with a high CA19-9 level.

## Figures and Tables

**Table 1:** Baseline clinicopathological characteristics.

Variable	More number group (*n* = 40)	Equal number group (*n* = 45)	*P* value
Age, years	53 (48, 63)	55 (47, 63)	0.905
Male (%)	31 (77.5)	28 (62.2)	0.127
CRC characteristics			
Rectum cancer (%)	11 (27.5)	12 (26.7)	0.931
T3/T4 stage (%)	38 (100)	38 (92.7)	0.089
N1/N2 stage (%)	31 (81.6)	17 (50.0)	0.005∗
TNM III/IV stage (%)	40 (100.0)	41 (91.1)	0.053
Resection with CRLM (%)	19 (47.5)	16 (35.6)	0.264
CRLM characteristics			
Preoperative chemotherapy (%)	37 (92.5)	45 (100)	0.061
Synchronous metastases (%)	36 (90.0)	31 (68.9)	0.017∗
CEA>200 *μ*g/L (%)	1 (2.6)	3 (6.7)	0.392
CA19-9 > 35 kU/L (%)	10 (25.6)	13 (28.9)	0.739
Tumor number in image	5 (2, 8)	5 (3, 8)	0.700
Tumor number in surgery	9 (5, 15)	5 (3, 8)	0.001∗
Tumor number in image >5 (%)	19 (47.5)	20 (44.4)	0.778
Tumor number in surgery >5 (%)	29 (72.5)	17 (37.8)	0.001∗
Tumor size, median (IQR)	1.7 (1-3.2)	2.5 (1.5-4)	0.085
R0 resection (%)	36 (90.0)	43 (95.6)	0.318
CRS score 3-5 (%)	25 (62.5)	15 (33.3)	0.007∗

Continuous variables are reported as the median and interquartile range. ^∗^*P* < 0.05. CRC = colorectal cancer; T stage = tumor stage; N stage = node stage; TNM stage = tumor node metastasis stage; CRLM = colorectal liver metastases; CEA = carcinoembryonic antigen; CA19-9 = carbohydrate antigen 19-9; IQR = interquartile range; R0 resection = hepatectomy on patients with clear histological margins; CRS = clinical risk score.

**Table 2:** Prognostic factors for overall survival and recurrence-free survival.

Variable	Overall survival				Recurrence-free survival			
	Univariate analysis		Multivariate analysis		Univariate analysis		Multivariate analysis	
	HR (95% CI)	*P* value	HR (95% CI)	*P* value	HR (95% CI)	*P* value	HR (95% CI)	*P* value
Age> 55 y	1.13 (0.48-2.67)	0.784			0.76 (0.42-1.37)	0.354		
Male	0.68 (0.28-1.62)	0.380			0.42 (0.23-0.77)	0.005		
Primary tumor								
Rectum cancer	0.87 (0.35-2.17)	0.762			0.56 (0.28-1.14)	0.112		
T3/T4 stage	0.76 (0.10-5.79)	0.789			1.50 (0.36-6.23)	0.581		
N1/N2 stage	1.98 (0.63-6.20)	0.240			1.16 (0.60-2.26)	0.658		
TNM III/IV stage	0.99 (0.55-1.77)	0.962			1.19 (0.79-1.80)	0.402		
Resection with CRLM	0.65(0.24-1.80)	0.410			0.85 (0.46-1.57)	0.608		
CRLM								
Synchronous metastases	1.13 (0.41-3.11)	0.817			1.12 (0.52-2.40)	0.781		
CEA > 200 *μ*g/L	1.25 (0.17-9.51)	0.827			1.11 (0.39-3.10)	0.850		
CA19-9> 35 kU/L	3.67 (1.55-8.70)	0.003^∗^	4.29 (1.77-10.37)	0.001^∗^	1.40 (0.74-2.60)	0.302		
Tumor number in image >5	1.12 (0.47-2.68)	0.799			1.27 (0.70-2.29)	0.436		
Tumor number in surgery >5	1.90 (0.78-4.63)	0.158			1.95 (1.07-3.56)	0.030^∗^	1.95 (1.07-3.56)	0.030^∗^
More tumor number than image	2.61 (1.08-6.28)	0.027^∗^	3.19 (1.28-7.97)	0.013^∗^	1.62 (-/88-2.97)	0.122		
Tumor size >5cm	4.36 (0.96-19.7)	0.056			1.57 (0.56-4.42)	0.392		
R0 resection	2.06 (0.66-6.41)	0.211						
CRS score 3-5	1.63 (0.68-3.91)	0.276			1.18 (0.65-2.15)	0.580		

HR = hazard ratio; CI = confidence interval; T stage = tumor stage; N stage = node stage; TNM stage = tumor node metastasis stage; CRLM = colorectal liver metastases; CEA = carcinoembryonic antigen; CA19-9 = carbohydrate antigen 19-9; R0 resection = hepatectomy on patients with clear histological margins; CRS = clinical risk score; ^∗^*P* < 0.05.

## Data Availability

The datasets generated during and/or analyzed during the current study are not publicly available due to hospital policy but are available from the corresponding author on reasonable request.
